# Transfer of abductor hallucis tendon combined with scarf osteotomy versus single scarf osteotomy in moderate to severe hallux valgus deformity: a comparative retrospective cohort study

**DOI:** 10.1186/s12891-019-2860-1

**Published:** 2019-10-20

**Authors:** Yuan Xiong, Bo Shen, Cheng Hao, Kai Xiao, Junwen Wang, Zhenhua Fang

**Affiliations:** Department of Orthopaedics, Wuhan Fourth Hospital, Puai Hospital, Tongji Medical College, Huazhong University of Science and Technology, Wuhan, 43033 China

**Keywords:** Hallux valgus, Musculus abductor hallucis tendon, Scarf osteotomy

## Abstract

**Background:**

Scarf osteotomy (SO) was broadly applied in moderate to severe hallux valgus (MSHV), and the results were satisfactory. However, due to the complicated pathologic changes in hallux valgus, the ideal surgical treatment is still controversial. Transfer of the abductor hallucis tendon combined with Scarf osteotomy (TAHTCSO) was an innovative alternative technique. This retrospective cohort study aimed to define if TAHTCSO mode resulted in improved outcomes as compared with the single SO in MSHV.

**Methods:**

Of 73 patients (92 ft) with MSHV, 36 (45 ft) were treated through TAHTCSO and 37 ones (47 ft) through SO. The patients were assessed clinically and radiographically with a 24-month follow-up. They were assessed pre-operatively and post-operatively with intermetatarsal angle (IMA), hallux valgus angle (HVA), distal metatarsal articular angle (DMAA), first metatarsophalangeal joint range of motion (1#MTP ROM), as well as American Orthopaedic Foot and Ankle Society (AOFAS) forefoot scores and postoperative complications of surgery.

**Results:**

Both cohorts had the same baseline feathures. All patients were followed up from 24 to 40 months, with a mean of 28.3 months. Patients in the TAHTCSO cohort had significantly decreased HVA at 6 months (*p* < 0.0001), 12 months (*p* < 0.0001), and 24 months (*p* < 0.0001) after surgery. 1#MTP had been increased slightly with non-statistic sense (*p***>**0.05). IMA, DMAA and AOFAS also had not significantly difference at all followed time after surgery as compared with the SO cohort. The healing of osteotomies was observed within 8 weeks in the two cohorts. Two cases of hallus varus had been found in SO cohort and there were no cases of delayed healing and bone non-union in both cohorts.

**Conclusion:**

In this retrospective cohort study, TAHTCSO had sufficient maintenance of the correction and improved functional performance thereby was a good alternative for MSHV, though it did not display a better result for MSHV compared to SO.

## Introduction

Moderate to severe hallux valgus deformity is defined as intermetatarsal angle (IMA) of more than 13 degrees [1]. It often occurs in the aged population with assessed occurrence of 36% in people over 65 years [2]. Some cases combined with other foot deformities, such as lesser toe, hindfoot or midfoot deformities may exacerbate the pathology. For MSHV correction, a variety of treatment options including foot orthoses, soft tissue release, and osteotomies are described [3–5]. However, the unique and most appropriate MSHV treatment remain elusive. The diaphyseal osteotomies such as Scarf osteotomy have gained popularity in recent years [6]. It provides excellent inherent biomechanical stability and allows early weight-bearing. In patients with MSHV, Scarf osteotomy seems to be a preferred surgery [7].

**Fig. 1:**
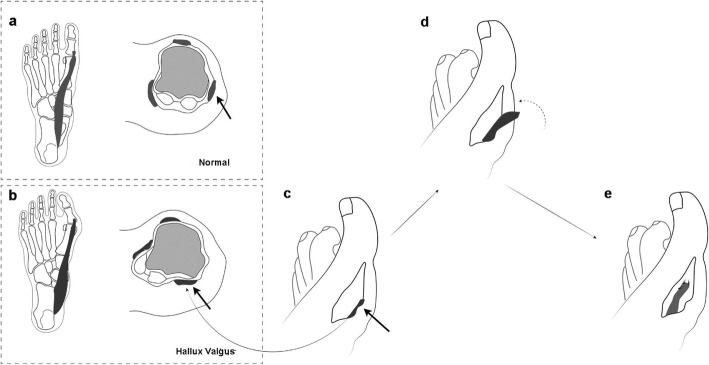
Transposition of abductor hallucis tendon. **a** the normal position of abductor hallucis. **b** abductor hallucis shift more plantar. **c**-**e** transposition of abductor hallucis tendon. Black arrow represents abductor hallucis

Abductor hallucis muscle plays an important role in maintaining first metatarsophalangeal joint stability and preventing abnormal transverse plane motion [8]. Transfer of the abductor hallucis tendon in hallux valgus surgery is a valuable addition in correcting the pronation deformity of the great toe [9]. TAHTCSO is an innovative alternative fixation approach (Fig. 1). This retrospective cohort study aimed to investigate if TAHTCSO results in improved outcomes as compared with single SO method.

## Materials and methods

### Patient eligibility

This is a retrospective study comparing the effects of TAHTCSO and SO in MSHV patients. From July 2013 to July 2015, 73 patients (92 ft) with MSHV were treated surgically and assessed retrospectively. Ethical approval and informed written consent were obtained from every single patient. Eligible patients were included in this research once they met the following criteria: (1) Diagnosed with MSHV (IMA more than 13 degrees), (2) were over 18 years of old and in full possession of their mental faculties, (3) had severe pain, (4) patients treated with TAHTCSO were included in TAHTCSO group or treated with SO were included in SO group, and were follow-up until 24 months, (5) first metatarsal osteotomy by a single surgeon, and (6) symptomatic hallux valgus associated with increased DMAA (≥15°).

### Surgical treatment and the rehabilitation protocol

**Fig. 2:**
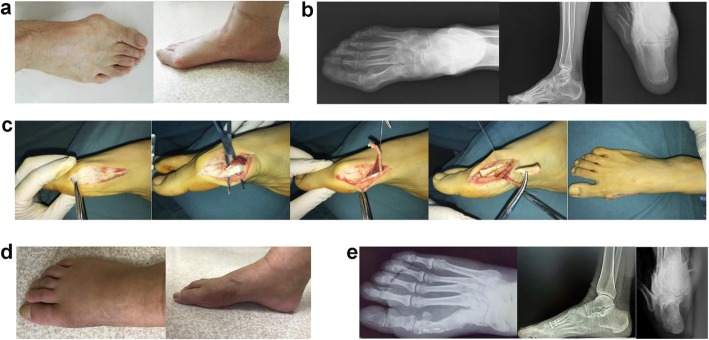
A 69–year-old woman had right-sided hallux valgus deformity. **a** to **b** Digital photos and X-ray showing the hallux valgus deformity. **c** The process of surgical technique. **d** Digital photos and X-ray after 24-month follow up

Surgically, all patients were treated by a single, senior surgeon for correcting deformity, relieving severe pain, and rebuilding inherent biomechanical stability. A medial incision is made, subcutaneous tissue was dissected bluntly in order to protect the medial dorsal neuro-vascular bundle. In TAHTCSO Cohort, the abductor hallucis tendon was be exposed, and 1/2~2/3 tendinous insertion of the abductor hallucis muscle was released from its ends and be transferred toward dorsal of the medial capsule, which can not only strengthen the medial tension but also achieve dynamic reconstruction of the medial soft tissue. The tendon was stitched with the capsule directly (Fig. 2). In both cohorts, patients were subjected to the same postoperative rehabilitation protocol, which allowed them to walk as much as they could on the heel side of the foot by a post-operative shoe after 3 days of surgery. Active and assisted motion of the metatarsophalangeal joint was permitted when suigical wounds were healed. Forefoot bandage and post-operative shoe were used for 4 and 6~8 weeks, respectively.

### Patient assessment

The baseline features comprising age, sex, BMI, and VAS pain score were collected. IMA, HVA, DMAA, and 1#MTP ROM were collected prospectively at predetermined intervals of 6, 12 and 24 months post-operatively. Subjective clinical results were measured through the AOFAS score which is one of the broadly applied clinician-reporting tools for forefoot and ankle illness. AOFAS is a clinician-based score that measures effects on four various anatomic regions of the foot: the anklehindfoot, midfoot, metatarsophalangeal (MTP)-interphalangeal (IP) for the hallux, and MTP-IP for the lesser toes. In our investigation, complications were also gathered until the 24 months’ follow-up.

### Statistical analysis

All statistical analyses were carried out via IBM SPSS Statistical software (version 22, IBM, Somers, NY, USA). The data was provided as the mean ± standard deviation (SD) for continuous variables and as numbers for categorical measures. The paired sample *t*-test was applied to compare the measurements before and after the surgery. *P*<0.05 was regarded as an important difference.

## Results

In this research, 36 patients (45 ft) treated with TAHTCSO and 37 ones (47 ft) treated with SO were taken part. The two cohorts had the same baseline features comprising mean age (65.9 + 10.4 and 66.8 + 10.5 years, *p* = 0.5132), gender distribution (male: female, n, 24:21 and 20:27, *p* = 0.0817), BMI (kg/m2, 27.4 + 3.9 and 29.0 + 6.4, *p* = 0.0526), and mean VAS pain score (87.2 and 92.9, *p* = 0.4133) (Table 1). Patients in the TAHTCSO cohort had significantly decreased HVA at 6 months (*p* < 0.0001), 12 months (*p* < 0.0001) and 24 months (*p* < 0.0001) after the surgery as compared with the SO cohort. Patients in the TAHTCSO cohort, had 1#MTP increased slightly with non-statistic sense (*p* **>** 0.05). IMA, DMAA and AOFAS also had not a significantly difference at all followed time after surgery as compared with the SO cohort. The healing of osteotomies was observed within 8 weeks in the two cohorts. Two cases of hallus varus and three cases of troughing were found in SO cohor; there were no cases of delayed healing and bone non-union in both cohorts (Table 2).

**Table 1:** The baseline characters of patients

	TAHTCSO(*n* = 45)	SO(*n* = 47)	*P* value
Age, y, mean + SD	65.9 + 10.4	66.8 + 10.5	.5132
Sex, male:female, n	24:21	20:27	.0817
BMI, kg/m2, mean + SD	27.4 + 3.9	29.0 + 6.4	.0526
VAS pain score (0–100), mean	87.2	92.9	.4133

*SO* Scarf osteotomy, *TAHTCSO* Transfer of the abductor hallucis tendon combined with Scarf osteotomy, *BMI* Body mass index, *VAS* Visual analog scale

**Table 2:** Clinical and radiographic assessment results of patients with MSHV

	TAHTCSO(*n* = 45)	SO(*n* = 47)	*P*	Test
IMA before surgery	16.47 ± 2.30	15.98 ± 1.89	0.3472	Unpaired T test
IMA 6 months	7.03 ± 0.70	7.27 ± 1.35	0.1405	Unpaired T test
IMA 12 months	7.31 ± 1.72	8.03 ± 3.67	0.2301	Unpaired T test
IMA 24 months	8.62 ± 2.49	9.02 ± 2.43	0.2505	Unpaired T test
HVA before surgery	34.02 ± 3.49	33.02 ± 2.43	0.9072	Unpaired T test
HVA 6 months*	11.96 ± 1.33	15.72 ± 3.99	< 0.0001	Unpaired T test
HVA 12 months*	13.03 ± 1.70	17.11 ± 3.69	< 0.0001	Unpaired T test
HVA 24 months*	13.71 ± 2.62	19.38 ± 2.53	< 0.0001	Unpaired T test
DMAA before surgery	9.71 ± 2.62	9.38 ± 2.53	0.4589	Unpaired T test
DMAA 6 months	6.09 ± 0.89	6.32 ± 0.77	0.0542	Unpaired T test
DMAA 12 months	6.23 ± 1.02	6.25 ± 1.15	0.1537	Unpaired T test
DMAA 24 months	7.05 ± 1.13	7.12 ± 0.89	0.2769	Unpaired T test
1#MTP ROM (dorsiflexion) before surgery	68.25 ± 2.90	68.73 ± 3.10	0.3681	Unpaired T test
1#MTP ROM (dorsiflexion) 6 months	69.23 ± 3.02	69.16 ± 1.89	0.1364	Unpaired T test
1#MTP ROM (dorsiflexion) 12 months	71.18 ± 2.81	70.09 ± 2.23	0.2246	Unpaired T test
1#MTP ROM (dorsiflexion) 24 months	73.78 ± 2.72	75.08 ± 1.87	0.3013	Unpaired T test
1#MTP ROM (plantarflexion) before surgery	33.05 ± 3.13	32.12 ± 2.89	0.2589	Unpaired T test
1#MTP ROM (plantarflexion) 6 months	35.72 ± 2.97	36.73 ± 1.78	0.1854	Unpaired T test
1#MTP ROM (plantarflexion) 12 months	36.63 ± 1.98	37.03 ± 1.89	0.2583	Unpaired T test
1#MTP ROM (plantarflexion) 24 months	37.73 ± 2.08	39.71 ± 1.06	0.2641	Unpaired T test
AOFAS before surgery	44.01 ± 0.56	43.24 ± 0.57	0.4709	Unpaired T test
AOFAS 6 months	76.67 ± 0.19	76.61 ± 0.15	0.3821	Unpaired T test
AOFAS 12 months	80.17 ± 2.15	81.64 ± 0.19	0.3531	Unpaired T test
AOFAS 24 months	80.69 ± 0.19	79.67 ± 0.39	0.1432	Unpaired T test
Complication hallus varus	0	2	0.0856	Fisher’s exact test
Complication delayed healing	0	0	–	–
Complication nonunion	0	0	–	–
Complication troughing	0	3	0.0512	Fisher’s exact test
Additional Akin osteotomy (n)	9	12	0.0631	Fisher’s exact test

*SO* Scarf osteotomy, *TAHTCSO* Transfer of the abductor hallucis tendon combined with Scarf osteotomy, *MSHV* Moderate to severe hallux valgus, *IMA* Intermetatarsal angle, *HVA* Hallux Valgus angle, *DMAA* Distal metatarsal articular angle, *MTP ROM* Metatarsophalangeal joint range of motion, *AOFAS* American Orthopaedic Foot and Ankle Society

* means *p* < 0.005

## Discussion

During recent years, the most commonly used alternative to MSHV is the SO that provides adequate contact between metaphyseal bone surfaces, prevents shortening of the first metatarsal and allows early mobilization [10, 11]. Adequate correction of the hallux valgus is obtained by greater rotation and translation of the plantar-distal fragment [12, 14]. Although SO is relatively easy for fixation, and our study did not find insufferable complications, it is technically demanding that can lead to worsening of symptoms if non-union or avascular necrosis of the first metatarsal head occurs [9, 11, 13]. In addition, the plantar fragment excessive rotation may result in an increase in the DMAA that can contribute to a lateral hallux deviation [8]. Although SO is identified by many surgeons for hallux valgus, it is far from a panacea [14]. Traditional SO simply releases the tendinous insertion of the adductor hallucis muscle and tightens the medial capsule. However, for MSHV with abnormal large HVA degree, strengthening the medial capsule is not enough to accomplish complete correction.

The major result of the current study is the efficacy of TAHTCSO for a novel approach. Abductor hallucis is located medial to the first metatarsal, which arises from the medial process of the calcaneal tuberosity and inserts on the medial surface of the base of the first proximal phalanx. It is the single muscle that directly may keep the hallux from abducting (displacing laterally) deformity [3]. In patients with hallux valgus, the abductor tendon shifts plantarward that contributes to the joint deformity [10, 12]. Despite the important role of the abductor hallucis muscle in the pathology of the deformity, few studies have examined the transfer of abductor hallucis muscle for hallux valgus correction [15]. The aim of the abductor hallucis transfer is to stabilize the medial soft tissue components of the first metatarsophalangeal joint [15]. In this study, part of the abductor hallucis tendon is released from its ends and transfered it toward dorsal of the medial capsule, which can not only strengthen the medial tension but also provide a dynamic reconstruction of the medial soft tissue. Transfer of the abductor hallucis tendon is important to restore the physiological situation and the first metatarsophalangeal joint function. This technique was recommended by our good clinical results in a whole follow up.

Akin osteotomy is often used to accompany scarf osteotomy. Kristen reported that Akin osteotomy is added after completing the scarf osteotomy and reconstructing soft tissue when the hallux is still greater than 10°of valgus position [7]. In other researches, Akin osteotomy has been reported to be used in 70 to 100% of cases after Scarf osteotomy [4, 6]. Unlike previous studies, only 9 ft were added an additional Akin osteotomy in TAHTCSO cohort. This may contribute to the fact that Scarf osteotomy combined with transfer of abductor hallucis tendon provides good correction for MSHV with no need to an Akin osteotomy; it suggested that the transfer of abductor hallucis muscle has strengthened the medial tension and developed a dynamic reconstruction of the medial soft tissue, and then can prevent HVA lost after operation. Troughing is another complication of Scarf osteotomy that takes place once the cortices wedge into the cancellous bone of the metatarsal shaft, which in turn result in stiffness of the first metatarsophalangeal joint [1]. Some studies found that it can be prevented by shifting the rotation of the osteotomy or transposition of the removed cortical bone between osteotomy sites [11, 13]. In TAHTCSO group, there was no troughing case, which may be contributed to the transposition of the removed cortical bone between osteotomy sites. Basically, the tendon transfer did not affect the troughing phenomenon. The troughing cases in TAHTCOSO may be contributed to some factors as below: 1. osteoporosis in patients; 2. the tendon transposition may intensify the HV correction ability and decrease the bone transposition to assure the cortical bone contraction. We also agree that TAHT is one of the most factors for causing iatrogenic varus. In present research, the reversed results may contribute to two main aspects. Firstly, although all the operations were performed by a same surgeon in this retrospective study, there were still some subtle differences in the intensity and degree of osteotomy among individual patients, which might lead to some differences that were not statistically significant. The other hand is that, the samples included in this study is limited, which is also the limitation of present retrospective study. In the follow-up study, we will further expand the sample size of the study, and throw high light on observing the differences of hallux valgus and other complications among groups.

Quantification analysis of the range of abductor hallucis tendon transfer has not been reported in previous studies. In the present study, 1/2~2/3 part of tendon of abductor halluces was released from its ending points and shifted towards dorsal of the first metatarsal capsule, which provided satisfying clinical result in the 24-month follow-up. However, for the long-term efficacy and potential complications (such as the residue abductor hallucis tendon rupture, and hallux varus), further studies are still needed to verify this surgical technique.

The strength of this study is that all surgeries were carried out by a single senior author. The clinical and radiographic data were assessed by three independent investigators respectively. The limitation of this study is that it was a retrospective study, with a few patients included. In addition, a 24-month follow-up can only be regarded as a short-term follow-up, which failed to provide information regarding the long-term consequences of the procedure. Furthermore, the patients with osteoporosis, osteoarthritis of the first metatarsophalangeal joint and so forth were excluded.

Above all, TAHTCSO is an effective technique for MSHV deformity. Although some cases with residual valgus need additional Akin osteotomies, all patients were satisfied with the function of their feet. The most important aspects that recommend the application of this method are its low number of complications, early weight-bearing, as well as good clinical and radiographic assessment results.

## Conclusions

In this retrospective cohort study, TAHTCSO had sufficient maintenance of the correction and improved functional performance, thereby a good alternative for MSHV, though it did not display a better results for MSHV as compared with SO.

## Data Availability

Please contact author Zhenhua Fang for data requests.

## Abbreviations

AOFAS: American Orthopaedic Foot and Ankle Society
DMAA: Distal metatarsal articular angle
HVA: Hallux Valgus Angle
IMA: Intermetatarsal Angle
MSHV: Moderate to severe hallux valgus
MTP ROM: Metatarsophalangeal joint range of motion
SO: Scarf osteotomy
TAHTCSO: Transfer of the abductor hallucis tendon combined with Scarf osteotomy
